# Finding balance between mature and immature neutrophils: The effects of empagliflozin in GSD‐Ib

**DOI:** 10.1002/jha2.649

**Published:** 2023-01-29

**Authors:** Fabiola Guerra, Serena Gasperini, Sonia Bonanomi, Viola Crescitelli, Roberta Pretese, Lorenzo Da Dalt, Giuseppe Danilo Norata, Marta Balzarini, Andrea Biondi, Andrea Baragetti, Francesco Saettini

**Affiliations:** ^1^ Department of Pediatrics Fondazione IRCCS San Gerardo dei Tintori Monza Italia; ^2^ Department of Pharmacological and Biomolecular Sciences University of Milan Milan Italy; ^3^ Pediatric Department ARNAS G. Brotzu Hospital Cagliari Italy; ^4^ School of Medicine and Surgery University of Milano‐Bicocca Milan Italy

**Keywords:** empagliflozin, glycogen storage disease type Ib, GSD‐Ib, G6PT, inborn errors of immunity, neutropenia, SLC37A4

To the Editor,

Glucose‐6‐phosphate (G6P) translocase (G6PT/SLC37A4) is an ubiquitously expressed enzyme and is required for the conversion of G6P to glucose, thus ensuring glucose production by the liver and kidney [1]. Biallelic loss of function in G6PT/SLC37A4 causes glycogen storage disease type Ib (GSD‐Ib), a rare disease with an incidence of ~1/100,000. GSD‐Ib is characterized by severe hypoglycemia, growth retardation, osteoporosis and long‐term risk of liver tumours and kidney failure [2].

In addition, individuals with G6PT deficiency also develop neutropenia, together with recurrent bacterial infections, gingivitis, periodontitis, genital and intestinal ulcers as a result of defective phagocytic function [1]. Neutropenia and neutrophil dysfunction in GSD‐Ib have been recently ascribed to the intracellular accumulation of 1,5‐anhydroglucitol‐6‐phosphate (1,5AG6P) that, by inhibiting the activity of hexokinases, limits the phosphorylation of glucose and derails the glycolytic pathway, essential for the immunometabolic activation and the patrolling activity function of these cells [3]. These findings set the stage for testing empagliflozin, an inhibitor of the kidney sodium glucose cotransporter 2 (SGLT2), which also lowers serum 1,5AG6P in GSD‐Ib patients. Data in G6PC3‐deficient mice and in a few G6PC3‐ or G6PT‐ deficient patients have shown that empagliflozin, by lowering serum 1,5‐AG and neutrophil 1,5 AG6P, improves neutrophil count and function [4].

This finding is in line with the deep connection between immune cell function and cellular metabolism [5]. Neutrophils change their demand of glucose over their maturation from immature to mature cells [6, 7]. Hence, it was speculated that empagliflozin, beyond the mere effect in increasing absolute neutrophil count (ANC), might promote the polarization toward specific neutrophil subtypes in patients affected by GSD‐Ib [8]. As mature neutrophils are co‐opted in lungs, gastrointestinal tract, and skin [7], investigating the differential neutrophil polarization following the treatment with empagliflozin in patients with G6PT deficiency may shed a light into the pharmacological benefit of the mechanism of gliflozins in GSD‐Ib patients [9].

To this aim, four GSD1b patients (9–16 years old), harboring biallelic *SLC37A4* variants and with ongoing granulocyte colony‐stimulating factor (G‐CSF) treatment, were enrolled for this in the study (Table 1). They were clinically evaluated at baseline and 3 months after empagliflozin treatment. Neutrophil subsets distribution in the peripheral blood at baseline and 3 months after empagliflozin were compared with those of eight age and sex matched healthy donors (HDs). Additional information is available in Supplementary Materials and Methods Section.

**TABLE 1:** Clinical characteristics of glycogen storage disease type Ib (GSD‐Ib) patients at basal evaluation and under empagliflozin treatment

	*P1, M, 10y*	*P2, M, 9y*	*P3, M, 11y*	*P4, F, 16y*
**Variants in SLC37A4**	c.92_94delTCT homozygous	c.902A > C, c.985‐2A > G heterozygous	c.1042_1043delCT homozygous	c.1124_del2 homozygous
**Empagliflozin final dose,** mg/kg/d	0.4 mg/kg/d	0.5 mg/Kg/d	0.6 mg/kg/d	0.5 mg/kg/d
**G‐CSF,** mcg/kg/wk	**BL**: 13,3, stop d31	**BL**: 19,6 stop d15	**BL**: 18, **UE**: 7,5	**BL/UE**: 45
**Median ANC,** 10^9^/L	**BL**: 1.59 **UE**: 2.16	**BL**: 4.4 **UE**: 3.14	**BL**: 0.32 **UE**:13.0	**BL**: 1.16 **UE**: 0.9
**Infections before empagliflozin treatment**	Recurrent otitis	Not reported	Sepsis, *Clostridium difficile* infection, pneumonia with right pleural effusion	Not reported
**Skin and mucosal lesions**	**BL/UE**: absent	**BL**: mucosal bleeding **UE**: absent	**BL**: recurrent aphthous stomatitis. **UE**: incidental, self‐limiting	**BL/UE**: absent
**IBD**	Not clinically suspected	Not clinically suspected	**BL**: mild **UE**: remission	**BL**: mild **UE**:remission
**Organomegaly** (ultrasound and physical examination)	**BL**: hepatosplenomegaly; **UE**: splenomegaly decreased	**BL/UE**: unchanged	**BL/UE**: unchanged	**BL/UE**: unchanged
**Clinically relevant hypoglycemia UE**	No	No	Yes (improvement of glycemic control)	Yes (improvement of glycemic control)

Abbreviations: ANC, absolute neutrophils count; BL, baseline; F, female; G‐CSF, granulocyte colony‐stimulating factor; M, male; UE, under empagliflozin treatment; Y, years old.

At baseline median ANC was 1.37 × 10^9^/L. Empagliflozin was gradually increased up to 0.4–0.6 mg/kg/day. G‐CSF administration was discontinued within the first month in two patients (P1 and P2). After 3‐months of treatment, median ANC increased to 2.56 × 10^9^/L and clinical improvement in all patients was noticed. P1 suffered from recurrent otitis and did not experience any further infection. P3 required multiple admission (i.e., *Clostridium difficile* infection, pneumonia with right pleural diffusion and sepsis) before empagliflozin treatment. He required significant G‐CSF dosage to maintain proper ANC (60 mcg/kg/wk).

Skin and mucosal lesions markedly decreased or were absent in P2 and P3. Inflammatory bowel disease (IBD) activity, measured by Ulcerative Colitis Activity Index and Pediatric Crohn's Disease Activity Index, improved from mild to under remission in two patients with active disease out of four patients (P3, P4). Organomegaly remained unchanged in all but P1, whose splenomegaly decreased. P3 and P4 reported hypoglycemia, effectively treated with diet‐modifications.

Out of four patients, three of them were immunophenotyped for neutrophils subsets, which were classified as mature (CD62L+CXCR2+), aged (CD62L‐CXCR2+), activated (CXCR4+CXCR2‐), immature (CD16+CD10^‐^), and pre‐neutrophils (CD49d‐CD101‐). Gating strategies are available in Supplementary materials and methods section (Figures S1 and S2). Baseline mature neutrophil levels were significantly lower in GSD‐Ib patients compared to HD (114 ± 106 cells/µL vs. 852 ± 98.1 cells/µLin HD) and increased following empagliflozin treatment (423 ± 246 cells/µL, *p* = 0.009) (Figure 1A). Although aged and activated neutrophils distribution did not significantly change (Figure 1B,C), we noticed increased activated neutrophils in two out of three children as compared to baseline (123–680 in P3 and 41 to 375 cells/µL in P2).

**FIGURE 1:**
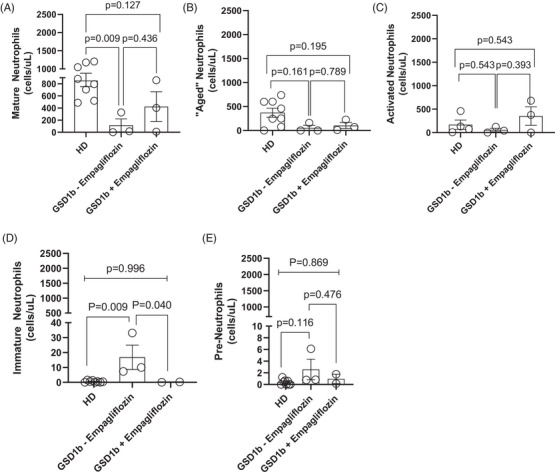
**Empagliflozin improves mature and reduces immature neutrophils form in glycogen storage disease type Ib (GSD‐Ib) patients**. Mature (A), aged (B), activated (C), immature (D) and pre‐neutrophils (E) at baseline and after 3 months of empagliflozin treatment in GSD‐Ib were compared to healthy donors

Immature neutrophils, which were higher in **GSD‐Ib** patients compared to HD (16 ± 8 cells/µL vs. 1 ± 0.2 cells/µL respectively, *p* = 0.009), significantly decreased following empagliflozin treatment (0.2 ± 0.1 cells/µL, *p* = 0.040 vs. pre‐treatment) with results comparable to HD (*p* = 0.996; Figure 1D). Empagliflozin did not affect circulating pre‐neutrophils (*p* = 0.869; Figure 1E).

Our data extend the existing knowledge regarding the efficacy of empagliflozin in GSD‐Ib, supporting that the beneficial clinical effect is not only due to the improvement of total ANC but could also results in the increase of specific neutrophil subtypes (mature neutrophils) and in the reduction of the immature subsets [8]. Our results confirm and extend the findings of Wortmann et al. [8], who proposed that the reduction in the occurrence of infections in G6PT deficient patients treated with empagliflozin could be related to the reduction of 1,5AG6P and to the improvement of glycolysis in mature neutrophils. Our data confirm Wortmann hypothesis, showing that the improvement in total ANC reflects in the increase of mature neutrophils and results in reduced infections as observed in the only patient in our cohort that had had recurrent infections.

Our data also show the reduction of immature neutrophils following treatment with empagliflozin, which have been found to be increased in G6PT patients [10]. Neutrophil dysfunction has been proposed as a main driver of inflammation in this condition. Recently, it has been reported that empagliflozin may reduce the burden of inflammatory conditions in GSD‐Ib patients [4, 8] and we observed an improvement in IBD in two patients, it is tempting to speculate that the benefit on

inflammation resulting from the treatment with empagliflozin n GSD‐Ib patients could be related to the reduction of this specific neutrophil subset, known to increase during either chronic and acute immunoinflammatory stresses [11].

In summary, our report reinforces the knowledge about the beneficial effects of empagliflozin in GSD‐Ib patients, highlighting, for the first time, that its clinical efficacy might also be linked to changes in the balance between mature and immature neutrophils. Larger multicenter studies with longer follow‐up are required to confirm these findings.

## AUTHOR CONTRIBUTIONS

FG, AB and FS contributed to conception and design of the study and wrote the first draft of the manuscript. AB performed neutrophils subsets analysis. All authors contributed to the article and approved the submitted version.

## CONFLICT OF INTEREST

The authors declare no competing interests.

## FUNDING INFORMATION

This research did not receive any specific grant from funding agencies in the public, commercial, or not‐for‐profit sectors.

## ETHICS STATEMENT

The studies involving human participants were reviewed and approved by the Fondazione MBBM, Monza, Italy. Written and informed consent to participate in this study was provided by the participants’ legal guardian/next of kin.

## Supporting information

Supporting information

## Data Availability

The raw data supporting the conclusions of this article will be made available by the authors, without undue reservation.
