# Supplementary material for: Finding balance between mature and immature neutrophils: The effects of empagliflozin in GSD‐Ib
Source: EJHaem. 2023 Jan 29;4(2):551–4. doi: 10.1002/jha2.649 (PMC10188444; doi:10.1002/jha2.649)
Supplement: Supplementary file 1 — Supporting information [file JHA2-4-551-s001.docx]

**Finding balance between mature and immature neutrophils: the effects of empagliflozin in GSD-Ib**

Guerra F^1^, Gasperini S^1^, Bonanomi S^1^, Crescitelli V^1^, Pretese R^1^,Da Dalt L^2^, Norata GD^2^, Balzarini M^3^, Biondi A^1,4^, Baragetti A^2^*, Saettini F^1^*.

^1^ Department of Pediatrics, Fondazione IRCCS San Gerardo dei Tintori, Monza, Italia

^2^ Department of Pharmacological and Biomolecular Sciences, University of Milan, Milan, Italy

^3^ Pediatric Department, ARNAS G. Brotzu Hospital, Cagliari, Italy;

^4^ School of Medicine and Surgery, University of Milano-Bicocca

**Co-corresponding authors:**

**Dr. Andrea Baragetti, PhD**

Department of Pharmacological and Biomolecular Sciences, University of Milan, Milan, Italy

Via G. Balzaretti, 9, 20133, Milan, Italy

Phone: +39 0250318401

Email: andrea.baragetti@unimi.it

**Dr. Francesco Saettini, MD**

Department of Pediatrics, Fondazione IRCCS San Gerardo dei Tintori, Monza, Italia

Via Cadore 26, 20900 Monza, Italy

Phone +39 0392333529

Email: f.saettini@gmail.com

**Supplemental materials and methods**

**Subjects for the study, clinical data and ethical aspects.**

Demographics information, clinical and laboratory data from medical case notes were collected. Complete blood counts, clinical course (infections, gastrointestinal symptoms, skin and mucosal lesions) at baseline and under empagliflozin administration were analyzed. The study conformed to the Declaration of Helsinki and was approved by the institutional review boards/ethic committee of Comitato Etico Brianza (Monza, Italy; GENPAT-PID). All patients, parents or legal guardians gave informed consent.

Healthy donors (HD) were enrolled from Progressione delle Lesioni Intimali Carotidee (PLIC) Study. PLIC is a large survey of the general population of the northern area of Milan (n = 2606) followed at the Center for the Study of Atherosclerosis, Bassini Hospital (Cinisello Balsamo, Milan, Italy). The Study was approved by the Scientific Committee of the Università degli Studi di Milano (“Cholesterol and Health: Education, Control and Knowledge—Studio CHECK (SEFAP/Pr.0003)—reference number Fa-04-Feb-01) on February 4th, 2001. An informed consent was obtained by participants of the study in accordance with the Declaration of Helsinki.

**Neutrophils characterization and Flow-cytometry**

Absolute neutrophils count (ANC) in whole blood and, out of this, the distribution of neutrophils subsets by Flow-Cytometry at basal evaluation and after 3 months of treatment were quantified.

“Mature” neutrophils were defined as CD62L+CXCR2+, “aged neutrophils” as CD62L-CXCR2+, “activated” neutrophils as “CXCR4+CXCR2-“ (see **Supplemental Figure 1** for detailed gating strategy). In a separate gating strategy, “Pre-neutrophils” were defined as (CD49d-CD101-) and “immature neutrophils” were defined as (CD16+CD10-) (see **Supplemental Figure 2** for detailed gating strategy).

All flow cytometry antibodies were used at 1:100 dilutions unless otherwise specified. 100 µL of blood were stained for the mixture of antibodies for the immunophenotyping of the neutrophils subsets. The different gating strategies for neutrophils subsets are presented in **Supplementary Figure 1**. After lysis of red blood cells and fixations (eBioscience™ 1-step Fix/Lyse Solution (10X) diluted 1:10 in distilled water (Cat#: 00-5333-54, ThermoFisher Scientific®), cell suspension was washed twice (Hank’s Balanced Salt Solution (HBSS) 1x, without Magnesium, without Calcium, without HCO_3_^-^ and without phenol red). Cells were re-suspended in 250 uL of HBSS and were then acquired with Novocyte 3000 Flow Cytometry platform (ACEA Biosciences). Absolute cell count was expressed as cells/microliter by normalizing the total number of events for the 100 uL acquired from the 250 microliter cell suspension. Finally, for 3 out of 4 patients we isolated and stored in liquid nitrogen circulating neutrophils by density-gradient centrifugation (density gradient with Histopaque^TM^ 1077 and 1.119 g/ml) for future cellular characterizations.

**Supplemental Figure 1. Flow-cytometry Gating strategy for the quantification of mature, aged and activated neutrophils.**

**
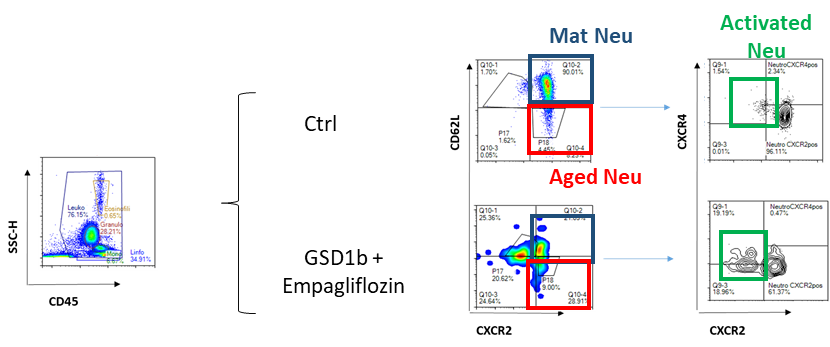
**

**Supplemental Figure 2. Flow-cytometry Gating strategy for the quantification of Pre-neutrophils, and Immature neutrophils.**

**
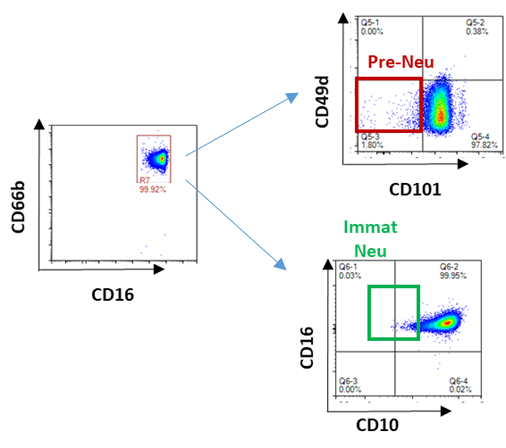
**

**Supplemental Table 1.** List of antibodies used for the gating strategies.

| **Antibodies** | **Source** | **Identifier** |
| --- | --- | --- |
| Human monoclonal anti-CD45 APC/Cy7 | BD Pharmingen® | Cat#: 557833; clone: 2D1 (RUO) |
| Human monoclonal anti-CD3 PerCP | Biolegend® | Cat#: 300428; clone: UCHT1 |
| Human monoclonal anti-CD34 APC | BD Pharmingen® | Cat#: 345804; clone: CE_IVD |
| Human monoclonal anti-CD16 PE | BD Pharmingen® | Cat#: 555407; clone: 3G8 (RUO) |
| Human monoclonal anti-CD11b FITC | Biolegend® | Cat#: 101206; clone: M1/70 |
| Human monoclonal anti-CD62L Brilliant Violet-650 | Biolegend® | Cat#: 304832; clone: MOPC-21 |
| Human monoclonal anti-CD182 (CXCR2) PE-Cyanine7 | Biolegend® | Cat#: 320716; clone: MOPC-21 |
| Human monoclonal anti-CD184 (CXCR4) Brilliant Violet-421 | Biolegend® | Cat#: 306518; clone: 12G5 |
| Human monoclonal anti-CD45 Alexa/Fluor-700 | BD Pharmingen® | Cat#: 560566; clone: HI30 (RUO) |
| Human monoclonal anti-CD66b PE/Cyanine7 | Biolegend® | Cat#: 305116; clone: G10F5 |
| Human monoclonal anti-CD10 Brilliant Violet-510 | Biolegend® | Cat#: 312220; clone: HI10a |
| Human monoclonal CD101 Alexa Fluor-647 | Biolegend® | Cat#: 331010; clone: BB27 |
| Human monoclonal anti-CD3 FITC | BD Pharmingen® | Cat#: 566783; clone: OKT3 (RUO) |
